# Integrated care for comorbid alcohol dependence and anxiety and/or depressive disorder: study protocol for an assessor-blind, randomized controlled trial

**DOI:** 10.1186/1940-0640-8-19

**Published:** 2013-11-19

**Authors:** Kirsten C Morley, Andrew Baillie, Claudia Sannibale, Maree Teesson, Paul S Haber

**Affiliations:** 1 National Health and Medical Research Council (NHMRC) Centre of Research Excellence in Mental Health and Substance Use, Discipline of Addiction Medicine, University of Sydney, Sydney, NSW, Australia; 2 NHMRC Centre of Research Excellence in Mental Health and Substance Use, Department of Psychology, Macquarie University, Sydney, NSW, Australia; 3 Discipline of Addiction Medicine, University of Sydney, Sydney, NSW, Australia; 4 Drug Health Services, Royal Prince Alfred Hospital, Sydney, NSW, Australia; 5 NHMRC Centre of Research Excellence in Mental Health and Substance Use, NDARC, Sydney, NSW, Australia

**Keywords:** Alcohol dependence, Anxiety, Depression, Cognitive behavioral therapy, Integrated care, Randomized controlled trial

## Abstract

**Background:**

A major barrier to successful treatment in alcohol dependence is psychiatric comorbidity. During treatment, the time to relapse is shorter, the drop-out rate is increased, and long-term alcohol consumption is greater for those with comorbid major depression or anxiety disorder than those with an alcohol use disorder with no comorbid mental disorder. The treatment of alcohol dependence and psychological disorders is often the responsibility of different services, and this can hinder the treatment process. Accordingly, there is a need for an effective integrated treatment for alcohol dependence and comorbid anxiety and/or depression.

**Methods/Design:**

We aim to assess the effectiveness of a specialized, integrated intervention for alcohol dependence with comorbid anxiety and/or mood disorder using a randomized design in an outpatient hospital setting. Following a three-week stabilization period (abstinence or significantly reduced consumption), participants will undergo complete formal assessment for anxiety and depression. Those patients with a diagnosis of an anxiety and/or depressive disorder will be randomized to either 1) integrated intervention (cognitive behavioral therapy) for alcohol, anxiety, and/or depression; or 2) usual counseling care for alcohol problems. Patients will then be followed up at weeks 12, 16, and 24. The primary outcome measure is alcohol consumption (total abstinence, time to lapse, and time to relapse). Secondary outcome measures include changes in alcohol dependence severity, depression, or anxiety symptoms and changes in clinician-rated severity of anxiety and depression.

**Discussion:**

The study findings will have potential implications for clinical practice by evaluating the implementation of specialized integrated treatment for comorbid anxiety and/or depression in an alcohol outpatient service.

**Trial registration:**

ClinicalTrials.gov Identifier: NCT01941693

## Background

There is a high rate of psychological comorbidity in people with alcohol dependence [1,2]. This is of concern given that, during treatment, the time to relapse is shorter, the drop-out rate is increased, and long-term alcohol consumption is greater for those with comorbid major depression or anxiety disorder than for those with an alcohol use disorder (AUD) with no comorbid mental disorder [3-5]. In the Australian National Survey of Mental Health and Wellbeing, Teesson and colleagues [6] reported that 33% of more than 10,000 respondents with an AUD also suffered from an anxiety or mood disorder as defined by the *Diagnostic and Statistical Manual of Mental Health Disorders,* 4th ed. (DSM-IV). In addition, this subpopulation was significantly more disabled and used health services more frequently than those with AUD with no comorbid mental disorder [6]. Results from the large alcohol treatment trial Project MATCH (Matching Alcohol Treatment to Client Heterogeneity) highlight psychiatric comorbidity as a significant factor influencing treatment response for alcohol dependence [7]. Similarly, we have reported that, among alcohol-dependent patients, clinically significant levels of depression predicted poor response to alcohol treatment [8]. Unfortunately, however, the treatment of alcohol dependence and psychological disorders is often the responsibility of different services, and this may hinder the treatment process. Accordingly, there is a need for an effective integrated treatment for alcohol dependence and comorbid anxiety or depression in outpatient services.

To date, research has usually assessed different psychosocial methods or pharmacological treatments targeted at alcohol dependence and psychological disorders separately. The focus has rarely been on concurrent treatment for both conditions. Regarding alcohol dependence and comorbid depression, there have been only two psychosocial intervention trials. Brown et al. [9] observed in 35 inpatients that adding CBT for depression versus relaxation training to standard partial hospital alcohol treatment was more effective in reducing depressive symptoms and some drinking outcomes than treatment for the alcohol problem only. They also observed that decreases in somatic depressive symptoms mediated the relationship between treatment condition and drinking outcomes. However, in a follow-up larger clinical trial, Brown et al. [10] found no significant differences on alcohol use outcomes and inconsistent effects on symptoms of depression. In both these studies, the interventions for depression and alcohol were not integrated, and the alcohol psychoeducational intervention was group-delivered only during the initial hospital stay.

There have been some studies investigating concurrent, but not integrated, treatments for alcohol dependence and anxiety disorders. Two studies found little beneficial effect from concurrent treatment for alcohol dependence and anxiety disorders (panic disorder and social phobia) [11,12]. However, in both studies, limited success may have been due, in part, to an inability to differentiate alcohol-related anxiety from non-alcohol–related symptoms at baseline assessment. Patients were either in withdrawal or not necessarily abstinent at the time of assessment, such that that anxiety symptoms resulting from alcohol withdrawal may have confounded the initial diagnoses. In many cases, symptoms of depression and anxiety resolve with abstinence so that further treatment is not required. However, in other cases where abstinence is achieved, these symptoms persist or worsen. To this degree, the stepped-care model entails assessing the outcome of primary treatment and systematically offering a secondary treatment where indicated and thus has the potential to provide a clearly structured, logical, and economical treatment [13-15].

More recently, Schadé et al. [16] trialed an intensive 32-week psychosocial and disulfiram treatment intervention for alcohol, either alone or in combination with CBT and optional pharmacotherapy for anxiety, among abstinent alcohol-dependent patients with comorbid social phobia or agoraphobia. They found that additional therapy for anxiety significantly reduced anxiety symptoms and avoidance behavior but did not affect alcohol relapse rates. With regard to post-traumatic stress disorder (PTSD), while reductions in alcohol consumption have reportedly mediated PTSD responsiveness [17], early improvement in PTSD symptoms appears to have a greater impact on improvement in alcohol dependence than the reciprocal relationship, which has prompted recommendations for integrated treatment [18]. To this degree, Mills et al. [19] examined integrated prolonged exposure treatment versus usual care for comorbid PTSD and substance dependence and observed a reduction in PTSD severity but no group differences in changes to substance use, depression, or anxiety. Sannibale et al. [20] recently investigated whether combining existing CBT interventions for AUD and PTSD would produce better outcomes than treating AUD alone. Although large overall reductions in PTSD and alcohol-related outcomes were found between groups, secondary analyses revealed a two-fold greater clinically significant change in PTSD severity for integrated therapy participants who attended one or more sessions of exposure therapy relative to participants treated for AUD alone.

Interestingly, Brown et al. [9,10] observed changes in the benefit of CBT for depression across two trials depending on the treatment context: they observed that CBT was more effective relative to a relaxation control when given separately with CBT for alcohol dependence in an inpatient versus an outpatient setting. The authors concluded that a synergistic beneficial treatment effect occurred in the inpatient setting due to CBT for depression being concurrently provided with CBT for alcohol dependence, albeit delivered by separate clinicians. This suggests the need to test the effectiveness of integrated interventions in specific service-delivery contexts.

One difficulty in devising integrated treatment for comorbid disorders is the limited research identifying reciprocal relationships between the disorders and the processes that may underlie these relationships. A number of processes have been proposed to underlie comorbidity between anxiety, depression, and alcohol dependence including 1) “self-medicating” a mood or anxiety disorder with alcohol [21,22], 2) the arousing and depressant properties of alcohol causing symptoms similar to anxiety and depression, and 3) trait-like factors such as anxiety sensitivity leading to poorly tolerated withdrawal [23,24]. Targeting specific mechanisms that may underlie comorbidity during treatment is likely to be a productive strategy.

We developed an integrated treatment for alcohol dependence and comorbid anxiety and/or mood disorder to be implemented and evaluated in an outpatient service-delivery setting. The specific aims of this study are 1) to assess the effectiveness of an integrated intervention compared with usual care at reducing alcohol consumption and anxiety and depression symptoms among alcohol-dependent patients with comorbid anxiety and/or depression in an outpatient hospital setting; 2) to determine factors that mediate the relationship between treatment condition and drinking and/or mood outcomes, and 3) to describe important factors relating to the maintenance of alcohol-related psychiatric comorbidity. This study design will draw on the stepped-care approach to interventions for alcohol dependence [14,15] and apply this approach to comorbid anxiety and depression by providing additional care for a second diagnosis when that diagnosis becomes clear. Hence, alcohol-dependent outpatients will undergo a stabilization period from alcohol followed by a formal assessment for diagnosis of anxiety and/or depression before being randomly allocated to receive either usual or integrated care.

## Methods

### Procedure

**Figure 1:**
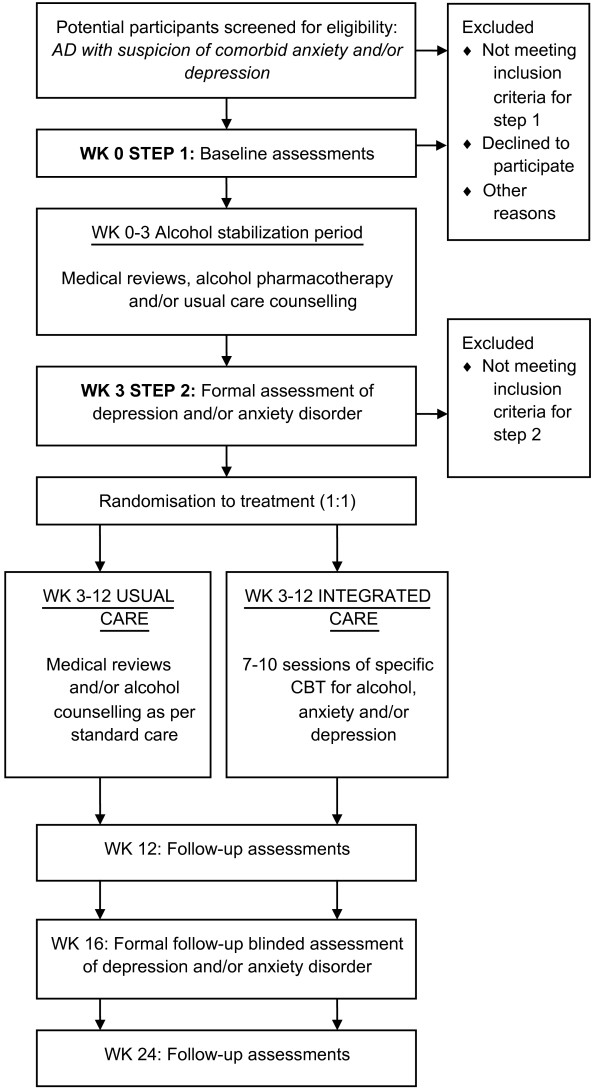
Flowchart of participants through the trial.

The flow of participants is outlined in Figure 1. The first phase of the study is to establish a stabilization period from alcohol for three weeks before entering the randomization step of the study. During the stabilization period, participants will have the option of pharmacotherapy using naltrexone (50 mg, 1 tablet daily), acamprosate (333 mg, 2 tablets 3 times daily, reduced to 4 per day for women weighing < 65 kg), or a combination of the two as medically prescribed based on physician judgment and participant preference. After a three- to four-week stabilization period, patients will undergo complete formal assessment for anxiety and depression. Those with a diagnosis of anxiety or depressive disorder, regardless of drinking outcome, will be offered the next step of care. The remaining participants will be monitored for a further nine weeks while receiving alcohol pharmacotherapy and/or usual counseling care. Patients to undergo the next phase will also continue to receive further alcohol pharmacotherapy as medically prescribed but will be randomized to one of two treatment groups: 1) integrated intervention for comorbid alcohol, anxiety, and/or depression, or 2) usual counseling care. Follow-up appointments will then be scheduled at weeks 12, 16, and 24 as described below.

### Participants

A total of 100 patients will be recruited from inpatients and outpatients of participating hospitals via flyers and community advertisements at local general practice offices, in newspapers, and on the Sydney Alcohol Treatment Group website. Conservatively assuming that 40% of respondents will not be eligible for randomization at step 2 following diagnosis for depression or anxiety disorder, we anticipate that 60 patients will be randomized to one of the two groups. Participant time and travel expenses will be reimbursed at follow-up assessments. The trial will be conducted at the Drug Health Services outpatient clinic of the Royal Prince Alfred Hospital, NSW, Australia. Ethics approval for the study has been granted by The Sydney Local Health District Ethics Review Committee (X05-0275).

### Randomization and allocation concealment

Randomization will be stratified according to concomitant selective serotonin reuptake inhibitor (SSRI) use and will by referring to the consecutively assigned subject identification number to a matched numbered envelope containing a random assignment card. Assessors will be blind to treatment allocation for the follow-up diagnostic interviews, and the same assessor will not assess the participant they assessed at baseline. Participants were asked not to mention their therapist or details of their therapy during their follow-up assessment. In order to evaluate the blinding process, assessors will be asked to which treatment group they believe each participant was allocated. A paper wall will be implemented whereby the researchers that obtain follow-up data will have no knowledge of treatment-group allocation.

### Step 1: inclusion and exclusion criteria for study enrollment

Inclusion criteria are as follows: alcohol dependence according to DSM-IV criteria, with alcohol as the subject’s drug of choice; age 18–65; adequate cognition and English-language skills to give valid consent and complete research interviews as assessed by the Mini Mental State Examination (MMSE); willingness to give written consent; abstinence from alcohol for between three and 21 days (standard clinical criteria for use of acamprosate or naltrexone); resolution of any clinically evident alcohol withdrawal as measured by the Clinical Institute Withdrawal Assessment for Alcohol–Revised (CIWA-Ar) [25]; and a positive score on the initial Comorbidity Suspicion Checklist (CSC). The CSC is a brief assessment to be administered at the first appointment. It is comprised of a one-page checklist to be filled in by one of the assessing clinicians to screen for current anxiety/depressive symptoms, previous diagnoses of comorbid conditions (anxiety disorder or depression), a previous history of treatment for comorbid conditions (anxiety disorder or depression), and a positive score on either the Mini Social Phobia Inventory (Mini-SPIN) for social anxiety [26] or the Kessler Psychological Distress Scale (K10) measure of psychological distress [27] for anxiety and depression.

Exclusion criteria include active major psychiatric disorder associated with significant suicide risk, pregnancy or lactation, advanced liver disease (hepatocellular failure, variceal bleeding, ascites, or encephalopathy), or other serious medical illness that would interfere with adherence to the study protocol.

### Step 2: inclusion and exclusion criteria for randomization

Inclusion criteria for randomization include abstinence and/or clinically significant reduction in alcohol use per clinician judgment, resolution of any clinically evident alcohol withdrawal as indicated by CIWA-Ar results, and case formulation and diagnosis for anxiety or depression. Exclusion criteria include alcohol consumption at baseline levels and resolution of clinically evident anxiety or depression as assessed by the case formulation (see below). These patients will be offered further treatment as appropriate within the service and will continue to be monitored.

### Instruments

#### Baseline assessment

Baseline assessment will be of 40 minutes’ duration and will be conducted upon consent to enter the trial. Data will be gathered on demographics, long-term alcohol consumption, medical history of alcohol- and nonalcohol-related illness, drug abuse, age of onset, and family history of alcohol problems. Recent (past 30-day) alcohol consumption will also be assessed by the timeline follow-back (TLFB) method [28]. Severity of dependence will be assessed by the Obsessive-Compulsive Drinking Scale (OCDS) [29] and by the Alcohol Dependence Scale (ADS) [30]. Functional disability will be assessed by the Short-Form Health Survey (SF-12) [31]. Depression, anxiety, and stress levels will be measured by the Depression Anxiety Stress Scales (DASS) [32]. Motivation to change and perceived self-efficacy are likely to vary between individuals and be a determinant of treatment outcome; therefore, these will be assessed by the Stages of Change Readiness and Treatment Eagerness (SOCRATES) Scale [33] and the Alcohol Abstinence Self-Efficacy (AASE) Scale [34]. Sleep disturbance will be assessed by the Insomnia Severity Index (ISI) [35], and the psychological basis of insomnia will be assessed by the Glasgow Sleep Effort (GSES) Scale. History of other drug use will be determined by the Opiate Treatment Index (OTI) interviewer-conducted questionnaire. Blood samples will be taken for full blood count and liver function tests.

#### Formal diagnosis of comorbid anxiety or depression (stepped-care assessment)

Several additional indices will be assessed at the commencement of the stepped-care phase:

•*Interview measures:* Recent (past 30-day) alcohol consumption will be available from the drinking diary cards offered upon entry to the trial. Diagnoses of anxiety and affective disorders will be established by the Anxiety Disorders Interview Schedule (ADIS) for DSM-IV [36]. The structured interview for the Hamilton Rating Scale for Depression (HRSD) will be used to measure the severity of depressive disorders [37]. The International Personality Disorder Examination [38] will be used to screen for personality disorders.

•*Questionnaire measures:* Continuous measures of change for anxiety or depression will be obtained by administering the DASS [32]. Recent severity of alcohol dependence will be assessed by the OCDS and by the ADS [30]. Functional disability will be assessed by the SF-12 [31]. Information from all sources will be integrated into a case formulation following methods described by Persons and Tompkins [39].

#### Follow-up schedule and assessments

Brief reviews will be scheduled with clinic medical staff at weeks 1, 6, 12, 16, and 24. Clinical and psychosocial events related to alcohol will be recorded along with serial liver function tests and mean corpuscular volume at baseline and at 6, 12, 16, and 24 weeks. At weeks 12, 16, and 24, the following research instruments will be administered as described above: DASS, ADS, SF-12, OCDS, GSES, ISI, AASE, SOCRATES, OTI, and a TLFB. At week 16, sections of diagnostic interviews relevant to diagnoses established at randomization (entry to step 2) will be readministered by a blind assessor. Recent alcohol consumption will be assessed as above and with a daily diary. Throughout the trial, any adverse and serious adverse events will be recorded. Participants will provide information concerning at least two contacts and receive three attempts at telephone or mail reminders of forthcoming appointments.

### Intervention

#### Usual care

Counseling for the treatment of alcohol dependence will continue in accordance with standard practice at the participating treatment site [40]. This entails brief individualized motivation enhancement therapy (ie, feedback of assessment findings, reinforcement, empathy, enhancing client’s own motivation). Participation in treatment sessions will be recorded. As quality-control measures, counselors will be supervised by senior staff and engage in weekly meetings, and treatment will be delivered according to the evidence-based treatment manual of Jarvis et al. [40].

#### Intervention for alcohol and comorbid anxiety and/or mood disorder

Structured interviews will be audiotaped and reviewed by the clinical team to develop consensus case formulations. Trained therapists will deliver specific CBT interventions based on evidence-based treatment manuals for alcohol use, anxiety, and depressive disorders (Table 1). Cognitive restructuring and graded exposure or behavioral experiments are techniques that are common to CBT for most of these individual disorders. Cognitive restructuring involves helping patients identify the key beliefs they hold about themselves, the future, the outside world, and other people that maintain their drinking, anxiety, or depression and assisting them to dispute and develop more helpful alternative beliefs. Graded exposure and behavioral experiments involve the gradual and programmed confronting of feared situations and has been argued to be the single most successful technique for overcoming phobias. These are necessarily brief descriptions for therapy that are well-documented in the treatment manuals displayed in Table 1. These manuals will be adapted for use with an alcohol-dependent population, and cognitive-behavioral coping skills and motivational enhancement strategies for alcohol consumption will be used where appropriate [41,42]. We expect different and complex interactions between the comorbid disorders, because the patients targeted for this study will experience significant comorbidity. An individualized case formulation, which hypothesizes the factors that maintain each individual’s problems, will be constructed following assessment using standard methods [39] (Table 2). The interventions will be delivered in seven to 10 sessions. To ensure that therapists adopt and maintain the principles of the therapy as described in the manual, sessions with consenting subjects will be audiotaped for discussion with the therapist early in the study (n = 4 per therapist) and at random times later in the study (n = 4 per therapist).

**Table 1:** Cognitive behavioral therapy manuals forming the basis of integrated care for depression, anxiety, and alcohol use disorders

**Diagnosis**	**Manual**
Depression	Persons et al. [43]
Social phobia	Rapee [44] Rapee & Sanderson [45]
Panic disorder or agoraphobia	Andrews et al. [46]
Generalized anxiety disorder	Andrews et al. [47]
Post-traumatic stress disorder	Foa & Rothbaum [48], Najavits [49]
Alcohol use disorders	Monti et al. [41] Miller et al. [42]

**Table 2:** Schedule of assessments

**Measure**	**Schedule**
Mini Social Phobia Inventory (Mini-SPIN)	Intake screening
Kessler Psychological Distress Scale (K10)	Intake screening
Comorbidity Suspicion Checklist (CSC)	Baseline (step 1*)
DSM-IV assessed dependence	Baseline (step 1)
Alcohol Dependence Scale (ADS)	Baseline (step 1), Weeks 12, 16, 24
Obsessive Compulsive Drinking Scale (OCDS)	Baseline (step 1), Weeks 12, 16, 24
Stages of Change Readiness and Treatment Eagerness (SOCRATES) Scale	Baseline (step 1), Weeks 12, 16, 24
Alcohol Abstinence Self-Efficacy (AASE)	Baseline (step 1), Week 12, 16, 24
Short-Form Health Survey (SF-12)	Baseline (step 1), Week 12, 16, 24
Depression Anxiety Stress Scale (DASS)	Baseline, Week 3 (step 2**), 12, 16, 24
Insomnia Severity Index (ISI)	Baseline, Week 3 (step 2), 12, 16, 24
Glasgow Sleep Effort Scale (GSES)	Baseline, Week 3 (step 2), 12, 16, 24
Timeline Follow-back (TLFB)	Baseline, Week 3 (step 2), 12, 16, 24
Anxiety Disorders Interview Schedule (ADIS)	Week 3 (step 2), Week 16
Hamilton Rating Scale for Depression (HRSD)	Week 3 (step 2), Week 16

*Step 1 = week 0 preceding three-week alcohol stabilization phase.

**Step 2 = formal diagnosis for depression and/or anxiety disorder preceding randomization to usual care or integrated care.

### Outcome measures

Primary outcomes include time to consumption of any alcohol (lapse) as identified by self-reported alcohol consumption; time to relapse as ≥ 4 drinks for women, ≥ 6 drinks for men; amount of alcohol consumed (total abstinence and average consumption per drinking day). Secondary outcomes include improvement in depressive or anxiety symptoms (DASS) or alcohol dependence severity (ADS); biological markers of alcohol consumption at 6, 12, 16, and 24 weeks (carbohydrate-deficient transferrin, liver function, and mean corpuscular volume); episodes of alcohol-related harm (psychosocial, occupational, forensic, medical); and clinician-rated severity of anxiety and depression based on ADIS and HRSD.

### Statistical analysis

The modified intention-to-treat principle will be used such that all subjects who attend the first intervention session will be analyzed [50]. The success of randomization will be tested by comparing baseline characteristics of the study groups with potentially confounding variables included as covariables. Analysis of variance will be used to compare continuous variables. Categorical variables will be compared via chi-square test. The effect of treatment on time-related outcome measures such as lapse and relapse will be analyzed by life-table survival analysis. Binary linear generalized estimating equations (GEE), with autoregressive correlation matrices, will be employed to determine differences between integrated care and usual care groups on change in anxiety or mood symptoms. A series of regression analyses will be performed to assess the role of symptomatic change in alcohol consumption and vice versa. Last-point carried forward or multiple imputation will be used to determine the scores of participants who drop out or are lost to follow-up. One previous study [9] with a similar design (N = 35) demonstrated that combined CBT treatment of alcohol dependence and comorbid depression significantly improved alcohol use outcomes with a moderate effect size. Power analysis has thus been performed such that a sample of 60 subjects (30 per group) has 80% power of detecting a moderate difference between the two arms of care on alcohol use outcomes at alpha = 0.05.

## Discussion

The main aim of the current study is to assess the effectiveness of a CBT-integrated intervention for alcohol-dependent patients with comorbid anxiety and/or depression compared with usual care in reducing alcohol consumption and symptoms of anxiety and depression in an outpatient hospital setting. This study has several strengths, including a randomized controlled assessor-blind design that attempts to address a serious gap in the treatment evidence. However, one challenge may include generalizability of the results given the specialized integrated intervention.

In addition, the secondary aims of the study are to determine factors that mediate the relationship between treatment condition and drinking and/or mood outcomes and to describe important factors relating to the maintenance of alcohol-related psychiatric comorbidity. Targeting specific mechanisms that may underlie comorbidity during treatment is likely to be a productive strategy. However, a limitation of the current study may include the capacity to document these processes and mechanisms with adequate power.

The study findings will have potential implications for clinical practice by evaluating the implementation of specialized integrated treatment for comorbid anxiety and/or depression in an alcohol outpatient service. The results may, therefore, improve treatment outcomes for people with alcohol dependence by addressing fundamental barriers to treatment response. A clearer understanding of these issues is a prerequisite to devising and implementing appropriate clinical interventions.

## Competing interests

The authors declare that they have no competing interests.

## Authors’ contributions

KM made contributions to the design of the study and wrote the manuscript. AB made contributions to the design of the study and the development of the integrated CBT intervention. He will also deliver and supervise the CBT intervention. MT conceived of the study and contributed to the design of the study. CS made contributions to the conception and design of the study and will deliver the integrated CBT interventions. PH made contributions to the design of the study and will deliver medical treatment and supervise the implementation of the study. All authors read and approved the final manuscript.
